# Application of a target enrichment-based next-generation sequencing protocol for identification and sequence-based prediction of pneumococcal serotypes

**DOI:** 10.1186/1471-2180-14-60

**Published:** 2014-03-10

**Authors:** Veranja Liyanapathirana, Irene Ang, Dominic Tsang, Kitty Fung, Tak Keung Ng, Haokui Zhou, Margaret Ip

**Affiliations:** 1 Department of Microbiology, The Chinese University of Hong Kong, The Prince of Wales Hospital, Hong Kong, China; 2 Queen Elizabeth Hospital, Hong Kong, China; 3 United Christian Hospital, Hong Kong, China; 4 Princess Margaret Hospital, Hong Kong, China

**Keywords:** Next generation sequencing (NGS), *Streptococcus pneumoniae*, Serotyping

## Abstract

**Background:**

The use of whole-genome sequencing in microbiology at a diagnostic level, although feasible, is still limited by the expenses associated and by the complex bioinformatics pipelines in data analyses. We describe the use of target enrichment-based next-generation sequencing for pneumococcal identification and serotyping as applied to the polysaccharide 23 valent vaccine serotypes as an affordable alternative to whole genome sequencing.

**Results:**

Correct identification of *Streptococcus pneumoniae* and prediction of common vaccine serotypes: 12 to serotype level and the rest to serogroup levels were achieved for all serotypes with >500 reads mapped against serotypes sequences. A proportion-based criterion also enabled the identification of two serotypes present in the same sample, thus indicating the possibility of using this method in detecting co-colonizing serotypes. The results obtained were comparable to or an improvement on the currently existing molecular serotyping methods for *S. pneumoniae* in relation to the polysaccharide vaccine serotypes.

**Conclusion:**

We propose that this method has the potential to become an affordable and adaptable alternative to whole-genome sequencing for pneumococcal identification and serotyping.

## Background

Identification and capsular serotyping of *S. pneumoniae* (pneumococcus) remains the cornerstone both for the diagnosis and surveillance of pneumococcal disease and carriage in the presence of extended spectrum pneumococcal conjugate vaccination. Currently available biochemical, serological and molecular methods are restricted by their throughput, turnaround time and limitations in multiplexing for multiple serotype detection [1,2].

Next Generation Sequencing (NGS) is a technique with the potential to fill this gap. Whole Genome Sequencing (WGS) on NGS platforms has been used for serotype prediction and for studying serotype changes in response to clinical interventions at research level [3,4]. In the diagnostic laboratory, WGS has been used to investigate potential outbreaks of drug-resistant pathogens [5]. However, the routine use of WGS with bench-top NGS remains limited by complex bioinformatics pipelines, computational capacities, relatively lower throughput per run without sample pooling, longer preparation times, and substantial costs.

Use of target enrichment followed by NGS has gained a wider use in cancer studies and the mutation analyses associated with congenital diseases [6,7]. The use of indexed sample preparation methods and pooling multiple samples lower the costs further. The combined use of target enrichment and sample pooling is gaining popularity as an option to sequence only the targets of interest at a higher depth of coverage enabling a more cost-effective use of sequencing reads; however, this strategy has hardly been explored in diagnostic microbiology. While the relatively smaller genome sizes in microbiology make the application of WGS attractive, adequate depth of sequences for interpretation may not be readily achievable. Target enrichment will enable a more cost-effective use of NGS in microbiology and overcome the issue of low copy numbers in the regions of interest. In this study, we explored a methodology based on target enrichment coupled with tagged sample pooling for the identification and serotyping of *S. pneumoniae* by using NGS. Criteria for the correct assignment of 23-valent polysaccharide vaccine (PPSV23) serotypes in relation to the total number of mapped reads per sample and the proportion of reads aligned to reference serotype sequences were also evaluated.

## Methods

### Bacterial isolates and DNA isolation

Twenty-four representative strains of *S. pneumoniae* of the PPSV 23 serotypes and serotype 6A, along with NCTC11189 *Streptococcus sp.* viridans type, obtained from the culture collection at the Department of Microbiology, The Chinese University of Hong Kong were used for the study. Isolates were grown overnight on blood agar plates. DNA was extracted by boil lysis, and the supernatant after centrifugation at 6,010 g for five minutes was used as the template. DNA quality and quantity of the neat and diluted samples were measured by the nanodrop method. In addition, multiple serotypes in varying proportions were also examined. Serotypes 19F and 3 were mixed in the proportion of 1:1, 1:10, 1:50, and 1:100 (1 = 75 ng/μl) to assess the feasibility of multiple serotype detection. DNA from NCTC11189 *Streptococcus sp.* viridans type was combined with that of serotype 19F at the proportions of 1:1, 1:10, 1:50, and 1:100 (1 = 75 ng/μl) to assess the feasibility of differentiating *S. pneumoniae* from members of the viridans group mimicking the nasopharyngeal niche.

### Targeted enrichment of serotype specific regions and sample pooling

A multiplex PCR using 23 previously described primer pairs [8], capable of amplifying the capsular genes of the polysaccharide vaccine serotypes (PPSV 23) and 17 closely related types (PCR 1), was used for target enrichment. An 18-bp nucleotide (nt) adaptor (TCTATTGGGCTATGTCAC) was incorporated to the 5’ end of each of the primers. The PCR-amplified fragments ranged from 259 to 413 bp in length.

A second multiplex PCR in addition to the abovementioned 23 primer pairs incorporated a pair of consensus primers (For- ATCGAACTCTTRCGYAATCTA and Rev-TCAAACTTRTCTTTTGGATAAGARC) targeting a sequence signature in the lytA gene capable of differentiating pneumococcus from the viridans group of streptococci (PCR 2).

Thermodynamic properties of the resultant primer adaptor combinations were evaluated by using the Oligoanalyzer 3.1 software (http://sg.idtdna.com/analyzer/applications/oligoanalyzer/). Eight 5-nt unique indexes were selected from the ready-made index sequences available at http://cloud.github.com/downloads/faircloth-lab/edittag/edit_metric_tags.txt[9]. One of these 5-nt indexes was attached to amplicons from each sample by way of a modified step-out PCR (MSO-PCR), so that the samples could be pooled prior to the library preparation [10]. The primer used for MSO-PCR had the same sequence as the 18-nt adaptor with unique 5-nt indexes at the 5’ end.

PCR 1 and 2 were performed by using the Platinum ® multiplex PCR kit (ABI by Life Technologies) with 1 μl of DNA extract as a template in a total reaction volume of 10 μl. The MSO-PCR had a total volume of 50 μl with 4 μl of PCR1 or PCR2 amplicons as templates and used Acti Taq DNA polymerase (Roche Diagnostics) for amplification. Thermocycling parameters were in accordance with the respective manufacturers’ instructions with annealing temperatures of 60°C and 53°C for 30- and 15-cycles, respectively.

### Sequencing and sequence analysis

PCR amplicons were purified by using a QIAquick PCR product purification kit (Qiagen) and quantified by using Qubit (Life Technologies). Eight samples tagged with unique indexes were pooled together at equal quantities to create a single sample for library preparation. Library preparation was performed by using TruSeq DNA library preparation kit V2 (Illumina) without fragmentation. Sequencing was performed on a Miseq bench-top sequencer (Illumina) with 2 × 150 bp methodology. Paired-end sequencing data from the Miseq reporter software was further analyzed off instrument. Quality filtering of the paired end data, de-multiplexing and trimming was performed by using a FASTX toolkit (http://hannonlab.cshl.edu/fastx_toolkit/) prior to mapping against reference sequences using Bowtie 2 [11]. The reference amplicon sequences for data alignment were generated from accession numbers cited by Kong *et al*. [8] for serotype-specific capsular gene sequences and [Genebank: AJ419979 and Genebank: AJ244307] for the atypical and typical pneumococcal lytA gene sequences, respectively.

### Data analysis

The experiments with multiple serotypes were designed to establish cut-off limits that would enable the identification of one or more serotypes from a sweep of colonies while excluding the possibility of false positive serotype allocation due to sample pooling. Thus, the percentage of reads assigned to each serotype out of the total number of mapped reads per given sample was calculated. The aim of PCR 2 that incorporated the lytA primers was to establish cut-off limits to identify pneumococci from previously unidentified α-haemolytic streptococci and to assign predicted serotypes. The percentage of reads assigned to the typical or atypical lytA genes out of the total mapped reads per sample and the percentage of the reads assigned to each serotype out of the total reads mapped against serotype sequences for the given sample were calculated. The percentages of correct serotype identification by using various criteria, including the proportion of mapped reads and a defined minimum number of total mapped reads, were examined.

## Results and discussion

The total numbers of mapped reads in the samples with a single serotype in the two reactions (PCR 1 and PCR 2) ranged from 169 to 4,987, with a median of 2,162 (IQR – 1,059, 3,691) reads, and 128 to 5,238, with a median of 1,658 (IQR – 807, 2,550) reads, respectively. Considering all samples, the mean percentage reads assigned to the correct serotype, per sample were 80.5% (95% CI 72.0–89.0%) and 80.7% (95% CI 72.9–88.5%) in the two PCR reactions, respectively. All serotypes but one was correctly assigned when the percentage reads matched to a single serotype exceeded 70% of all the reads in the sample.

The percentages of correctly identified pneumococcal serotypes based on various cut-off limits are listed in Table 1. The first criterion for serotype allocation was defined by the presence of >15% reads out of total reads mapped against serotype sequences per sample. The percentages of correct serotype identification based on this were 87.5% and 91.7% for PCR 1 and PCR 2, respectively. The cut-off of >15% reads was calculated based on the experimental evaluation of multiple serotypes (please refer to paragraph 3 of Results and Discussion). The percentage of correct identification increased when more stringent criteria were added in defining the cut-off. The percentage of correctly serotyped isolates reached 100% when only the samples with >500 reads mapped were considered for serotype allocation. The results interpretation for individual samples based on each of the three criteria; >15% mapped reads (criterion 1), addition of >200 (criterion 2), or >500 (criterion 3) reads mapped against the serotype sequences, are listed in Tables 2 and 3. Considering only criterion 1, in all five instances where serotypes were incorrectly assigned, the total number of reads mapped to the serotype sequences was <500. In PCR 1, both samples with aberrant results had a total number of mapped reads <200. For PCR 2, the correct identification of a single serotype was possible in 21/24 (87.5%) of all samples, 21/23 (91.3%) of samples with >200 reads, and 19/19 (100%) of samples >500 reads (Table 1). Thus, a minimum number of reads mapped against serotype sequences of >500 was considered as the criterion to filter samples suitable for serotype allocation.

**Table 1:** Percentage of correct identification of pneumococcal serogroup/serotypes based on different cut-off limits on number of reads

**Sample category**	Definition for interpretation cut-off*	**Median no. reads mapped against serotype/group (IQR)**	**Mean percentage of reads aligned against correct serotype (95% CI)**	**Mean percentage of reads aligned against second serotype (if present) (95% CI)**	**No. of samples**	**Percentage of correct ID**
PCR1 (serotype assignment only)	All samples^**^	2,162 (1,059–3,691)	80.5% (72.0–89.0%)	8.8% (2.7–14.9%)	24	91.7%
	Samples with >200 reads mapped against serotypes	2,229 (1,328–4,257)	86.2% (83.1–89.3%)	5.0% (3.7–6.34%)	23	100%
	Samples with >500 reads mapped against serotypes	2,245 (1,530–4,371)	86.5% (83.3–89.7%)	4.9% (3.5–6.3%)	21	100%
PCR2 (serotype and ID)	All samples^**^	1,658 (807–2,573)	80.7% (72.9–88.5%)	7.5% (4.3–10.7%)	24	87.5%
	Samples with >200 reads mapped against serotypes	1,683 (898–2,574)	83.0% (76.4–89.6%)	6.6% (3.8–9.4%)	23	91.3%
	Samples with >500 reads mapped against serotypes	1,951 (1,178–2,633)	87.1% (85.1–89.1%)	4.8% (2.3–7.3%)	19	100%

^*^Criteria for serotype allocation. Each category fulfilled the minimum criterion of >15% reads out of total reads assigned to the sample.

^**^Criterion of >15% reads out of total reads mapped assigned to the sample.

**Table 2:** Detailed results of samples with a single serotype for serotype assignment

**Sample ID**	**Serotype**	**Total reads mapped against serotype sequences**	**% reads mapped against correct serotype**	**% reads mapped against the second match**	**Serotype(s) identified**		
					Criterion 1*	Criterion 2**	Criterion 3***
1	1	4,711	91.9	3.0	1	1	1
2	2	2,245	76.8	8.9	2	2	2
3	3	160	0	75.0	Wrong allocation of serotype	Insufficient reads	Insufficient reads
4	4	4,985	77.2	10.3	4	4	4
5	5	915	96.1	1.3	5	5	5
6	6B	2,213	96.8	1.0	6	6	6
7	6A	1,241	91.8	3.9	6	6	6
8	7F	222	81.5	6.3	7A/F	7A/F	Insufficient reads
9	8	2,955	77.4	3.985	8	8	8
10	9V	4,823	75.5	14.1	9A/V	9A/V	9A/V
11	9N	4,987	88.7	3.0	9N/L	9N/L	9N/L
12	10A	4,257	81.4	6.9	10A/B	10A/B	10A/B
13	11D	1,703	84.9	5.9	11A/D	11A/D	11A/D
14	12F	5,625	94.7	1.9	12/44/46	12/44/46	12/44/46
15	14	2,110	88.3	3.1	14	14	14
16	15B	998	84.9	3.1	15B/C	15B/C	15B/C
17	17F	1,357	72.7	7.3	17F	17F	17F
18	18C	2,418	84.5	3.9	18	18	18
19	19A	3,124	82.0	7.7	19F	19F	19F
20	19F	2,864	87.7	4.9	19A	19A	19A
21	20	169	36.7	27.2	Serotypes 20 and incorrect identification of serotype 23F	Insufficient reads	Insufficient reads
22	22F	1,948	92.8	3.7	22F/A	22F/A	22F/A
23	23F	1,877	95.1	2.6	23F	23F	23F
24	33F	625	94.4	2.4	33F/A/37	33F/A/37	33F/A/37

*Criterion 1: Any serotype with >15% sequence reads mapped assigned to the sample.

**Criterion 2: Samples with >200 reads considered for further typing AND fulfilled criterion 1.

***Criterion 3: Samples with >500 reads considered for further typing AND fulfilled criterion 1.

**Table 3:** Detailed results of samples with a single serotype for pneumococcal identification and serotype assignment

**Sample ID**	**Serotype**	**Total reads mapped against serotype sequences**	**% reads mapped against correct serotype^**	**% reads mapped against the second match^**	**Serotype(s) identified**			**No of reads mapped for *S. pneumoniae *** **(typical lytA)**	**No of reads mapped for viridans streptococci (atypical lytA)**
					Criterion 1*	Criterion 2**	Criterion 3***		
25	1	776	81.2	7.2	1	1	1	2,073	1
26	2	271	32.5	23.6	2 and incorrect	2 and incorrect	Insufficient reads	4,123	0
					ID of serotype 6	ID of serotype 6			
27	3	1,127	78.9	8.8	3	3	3	4,646	0
28	4	416	39.2	31.3	4 and incorrect	4 and incorrect	Insufficient reads	4,595	0
					ID of serotype 1	ID of serotype 1			
29	5	1,181	92.6	5.0	5	5	5	1,048	0
30	6B	2,501	97.4	1.3	6	6	6	1,294	0
31	6A	898	92.7	3.0	6	6	6	490	0
32	7F	360	89.2	5.0	7A/F	7A/F	Insufficient reads	292	0
33	8	1,868	74.4	6.5	8	8	8	7,396	0
34	9V	2,095	83.2	4.5	9A/V	9A/V	9A/V	2,992	0
35	9N	5,238	91.7	2.5	9N/L	9N/L	9N/L	5,934	0
36	10A	4,341	82.0	7.8	10A/B	10A/B	10A/B	14,623	0
37	11D	1,951	85.3	7.0	11A/D	11A/D	11A/D	4,844	0
38	12F	5,224	92.6	1.7	12/44/46	12/44/46	12/44/46	5,881	0
39	14	1,968	88.2	3.6	14	14	14	4,624	0
40	15B	986	89.6	3.8	15B/C	15B/C	15B/C	1,184	0
41	17F	1,178	71.7	7.1	17F	17F	17F	5,387	0
42	18C	2,699	97.0	0.8	18	18	18	4,502	0
43	19A	2,644	82.2	7.7	19F	19F	19F	3,079	0
44	19F	2,598	87.1	5.7	19A	19A	19A	5,570	0
45	20	128	28.9	26.6	20 and incorrect	Insufficient reads	Insufficient reads	1,946	0
					ID of serotype 23F				
46	22F	1,683	92.5	4.2	22F/A	22F/A	22F/A	2,107	0
47	23F	1,633	94.2	2.1	23F	23F	23F	1,404	0
48	33F	475	93.5	2.1	33F/A/37	33F/A/37	Insufficient reads	496	0

^ Denominator - total number of reads mapped against serotype/group specific sequences.

*Criterion 1: Any serotype with >15% sequence reads mapped assigned to the sample.

**Criterion 2: Samples with >200 reads considered for further typing AND fulfilled criterion 1.

***Criterion 3: Samples with >500 reads considered for further typing AND fulfilled criterion 1.

Table 4 shows the results of individual samples containing two bacterial isolates tested for pneumococcal identifications and/or serotype assignment. These results of mixed serotypes were used to establish a minimum percentage of reads as a cut-off for allocating serotypes, and it enabled the identification of a second serotype. The numbers of reads mapped against a given serotype was not proportional to the original ratio of input DNA. The total number of reads mapped against serotype 3 was in general lower than that of serotype 19F. The mean proportion of reads mapped against serotype 3 was 18.4% (95% CI 16.0–20.8%) for PCR 1 (Table 4a) and 18.2% (95% CI 15.6–20.8%) for PCR2 (Table 4b). A cut-off of 15% based on the lower limit of total reads mapped to particular serotype sequence, was used for the assignment of a serotype. Accordingly, the result would not be analyzed where the cut-off or coverage threshold is not achieved [12]. Thus, we propose that samples with >500 reads assigned to serotypes should be considered for further serotype allocation and any serotype with >15% of the reads assigned should be considered as present within the sample. Applying both criteria, 22 samples tested in PCR 1 and 19 samples in PCR 2 were eligible for further analysis, and all these samples were allocated serotypes correctly.

**Table 4:** Detailed results of samples containing multiple bacterial isolates for pneumococcal identifications and/or serotype assignment

**Sample ID**	**Sample composition (ratio of serotypes)**	**Total mapped reads per sample**	**Total reads mapped against serotype sequences**	% reads mapped against serotype 19F*	% reads mapped against serotype 3*	% reads mapped against other serotype*	**Number of reads mapped against lytA sequences**	**% reads mapped for *S. pneumoniae *** **(typical lytA)**	**% reads mapped for other viridans streptococci (atypical lytA)**
**(a) Samples with two serotypes tested with PCR 1**									
49	1:1, 3:19F	16,318	16,318	77.8	22.1	0	NA	NA	NA
50	1:10, 19F:3	17,473	17,473	80.8	18.9	0.1	NA	NA	NA
51	1:10, 3:19F	19,239	19,239	85.0	14.8	0	NA	NA	NA
52	1:50, 19F:3	11,986	11,986	80.2	19.6	0	NA	NA	NA
53	1:50, 3:19F	4,853	4,853	82.3	17.5	0.1	NA	NA	NA
54	1:100, 19F:3	7,447	7,447	77.4	22.2	0.1	NA	NA	NA
55	1:100, 3:19F	4,251	4,251	85.5	13.7	0.2	NA	NA	NA
**(b) Samples with two serotypes tested with PCR 2**									
56	1:1, 3:19F	9,700	6,631	76.2	22.8	1.0	3,069	99.7	0.3
57	1:10, 19F:3	17,282	12,952	79.8	19.9	0.2	4,330	99.7	0.3
58	1:10, 3:19F	10,644	7,690	84.1	15.8	0.2	2,954	99.7	0.3
59	1:50, 19F:3	11,623	8,666	79.2	20.7	0.2	2,957	99.6	0.4
60	1:50, 3:19F	6,378	4,392	85.2	14.6	0.3	1,986	99.4	0.6
61	1:100, 19F:3	8,673	6,385	79.9	19.8	0.3	2,288	99.4	0.6
62	1:100, 3:19F	5,740	4,023	85.8	13.9	0.4	1,717	99.0	1.0
**(c) Samples with proportionately mixed *Streptococcus pneumoniae *** **and NCTC 11189 *Streptococcus sp. *** **viridans type. tested with PCR 2**									
63	1:1, NCTC 11189:19F	10,408	7,621	99.9	NA	0	2,787	46.6	53.4
64	1:10, 19F:NCTC 11189	3,459	2,112	99.9	NA	0.1	1,347	32.5	67.5
65	1:10, NCTC11189:19F	1,252	842	97.7	NA	1.8	410	86.1	13.9
66	1:50, 19F:NCTC 11189	18,079	10,467	100	NA	0	7,612	10.2	89.8
67	1:50, NCTC 11189:19F	16,336	10,172	99.8	NA	0	6,164	81.4	18.6
68	1:100,19F:NCTC 11189	18,259	8,612	100	NA	0	9,647	7.6	92.4
69	1:100, NCTC 11189:19F	8,694	3,559	100	NA	0	5,135	64.4	35.6

*Denominator – total number of reads mapped against serotype/group specific sequences.

NA = Not applicable.

PCR 2 enabled the confirmation of pneumococcal identification as well as the detection of the presence of a viridans group of streptococci. Although the proportions of the viridans group of streptococci and *S. pneumoniae* were not quantifiable, it is possible to apply this method to identify and serotype pneumococci from non-purified primary cultures of α-haemolytic colonies.

The inability of the method to quantify the proportion of serotypes or bacterial species within a sample could be due to amplification bias in the PCR efficiency of the primers and the differences in the target sequences. However, a second serotype could still be qualitatively identified.

The method used for quantification of DNA prior to pooling the samples could be improved in terms of specificity by using a fluorescence based quantification method and this may help to improve the results pertaining to quantified identification. However, as the measurement of DNA quality excluded possible protein contamination and as the outcome was measured in a qualitative manner instead of a quantitative manner, the impact on the final results was negligible.

The pooling of samples with the addition of unique nucleotide tags helped us to reduce the associated cost. However, this also introduces the possibility of false positive identification of a second serotype due to chimeric amplicon generation. Thus, to achieve a balance between detecting co-colonization and sample pooling the cut-off for defining serotypes needs to be fine-tuned for the given number of samples pooled and it could therefore be considered as a limitation of the method. Where the presence of a second serotype is a possibility, the cut-off determination needs to consider the possibility of including a false positive second serotype with a lower threshold and the false negative exclusion of a minor serotype with a higher cut-off limit.

If the method is to be applied to identified pneumococcal isolates where a single serotype is expected, the serotype with the highest proportion of reads mapped would be the only one found in the given sample. The use of PCR 1 for target enrichment is sufficient when the objective would be to identify the serotype of an identified pneumococcal isolate. The use of PCR 2 for target enrichment is recommended when the template is DNA from primary cultures or patient samples; as it identifies the presence of pneumococci by the characteristic lytA sequences. This would enable the identification of samples containing serotypes not included in the current PCR and samples with abnormal non-pneumococcal isolates harboring capsular sequences.

The protocol could potentially be extended to include more serotypes and direct detection from clinical samples. The validation process could be improved with the evaluation of the performance of different indexes and the establishment of the maximum plexity achievable for sample pooling. The whole process currently takes about 50 hours to complete; however, the method is amenable to automation and the recently released Truseq Nano (Illumina/USA) commercial test has potential to reduce the time required. Even with the current turn-around time, this protocol is appropriate where a larger number of samples are to be tested or if applied to direct sample testing. The analysis process could be further simplified by using the Custom Amplicon Workflow of Miseq reporter with alternative amplicon manifest created where the in-house index-adaptor-primer sequences could substitute the upstream and downstream probe sequences and a pseudo-genome created by all possible amplicon-adaptor-index combinations could be used instead of the reference genome.

As proof of concept, we demonstrated that target enrichment-based NGS could be applied with similar results to the conventional molecular serotyping methods. The serotype resolution for the PPSV 23 capsular types from this method is similar to that of the recently recommended triplex RT-PCR method by CDC, Atlanta, USA (http://www.cdc.gov/ncidod/biotech/strep/pcr.htm, accessed on 01/06/2013). However the latter method does not include serotypes 10A/F and 15B/C, and it requires seven sequential PCRs. A sequetyping method described by Leung *et al*. using a pair of consensus primers, although is simpler in terms of the number of primers used, does not achieve the same serotype grouping pattern as the one in the current protocol; furthermore, the success rate for samples of PPSV 23 serotypes was lower at 86% [13]. All molecular methods, including the current method, have the drawback of not being able to differentiate some very closely related serotypes.

A number of comparable molecular methodologies with the capability of detecting colonization of multiple serotypes have recently been described [14-17]. Direct clinical samples and the sweep of colonies from primary plates have been employed as the templates for these methods. The sequential application of multiplex PCR has been shown to detect multiple serotypes from nasopharyngeal secretions [14]. The currently widely used CDC-recommended multiplex PCR is capable of identifying 40 different sero-identities by eight multiplexes while in the current method, a single PCR is capable of enriching 23 polysaccharide capsular vaccine serotypes, at least to related serogroups in a single reaction. There is potential to expand the number of sero-identities in the current method because the resolution is not limited by the need for bands with detectable differences in lengths. Microarray-based serotyping methods have been used successfully to identify and type pneumococcal serotypes [15-17]. However, microarray technology remains expensive and it needs complex interpretations whereas the bench-top NGS technology is becoming more user-friendly [1]. PCR followed by capillary electrophoresis and PCR followed by ionization mass spectrometry with the capability of detecting multiple colonization, have also been described [18,19]. However, NGS with its competitive market is becoming more affordable and this is the first instance to the best of our knowledge that target enrichment coupled to NGS has been used to develop an assay to identify and serotype *S. pneumoniae*.

The routine use of WGS in a microbiology laboratory using the same platform has been evaluated recently [20]. However, the number of samples to be pooled together is limited. Our results demonstrate that target enrichment-based NGS could be used for the identification and sequence-based serotype prediction of pneumococcus. Development of proprietary chemistry-based methods for target enrichment is not widely available for microbiological applications and the kits remain expensive. However, the availability of *Taq polymerases* with increased multiplexing capabilities and the elimination of the necessity for the gel-based differentiation of bands increase the possibility of using multiplex PCR for target enrichment. In addition to using this method for typing organisms, syndrome-based identification panels could also be developed by using a similar methodology.

## Conclusions

We demonstrated the feasibility of using a custom enrichment-based sequencing methodology for *S. pneumoniae* identification and serotyping. A multiplex PCR containing primers for serotype/group specific regions of the 23 valent polysaccharide vaccine serotypes was used for target enrichment followed by NGS on a Miseq platform to serotype pneumococci successfully at cut-off read levels defined during the study. A second multiplex PCR containing an additional pair of primers that help to identify pneumococci was also successfully applied for target enrichment. The first enrichment PCR could be used to serotype identified pneumococcal isolates while the latter one could be used on primary cultures and direct samples. The principle of using simple multiplex PCR for target enrichment followed by NGS could be adapted for syndrome-based diagnosis and typing using either bacterial isolates or patient samples.

## Availability of supporting data

Not available.

## Competing interests

The authors declare that they have no competing interests.

## Authors’ contributions

VL, DT, KF, TN, and MI were involved in the design of study and the provision of strains for study. VL and IA performed the laboratory experiments. VL, IA, HZ, and MI were involved in data analyses. VL drafted the manuscript, and all members contributed to the preparation of the final manuscript. All authors read and approved the final manuscript.
